# Brain-Computer Interface using neural network and temporal-spectral features

**DOI:** 10.3389/fninf.2022.952474

**Published:** 2022-10-05

**Authors:** Gan Wang, Moran Cerf

**Affiliations:** ^1^School of Mechanical and Electrical Engineering, Soochow University, Suchow, China; ^2^Interdepartmental Neuroscience Program, Northwestern University, Evanston, IL, United States

**Keywords:** Brain-Computer Interfaces, motor, EEG, neural networks, deep learning

## Abstract

Brain-Computer Interfaces (BCIs) are increasingly useful for control. Such BCIs can be used to assist individuals who lost mobility or control over their limbs, for recreational purposes such as gaming or semi-autonomous driving, or as an interface toward man-machine integration. Thus far, the performance of algorithms used for thought decoding has been limited. We show that by extracting temporal and spectral features from electroencephalography (EEG) signals and, following, using deep learning neural network to classify those features, one can significantly improve the performance of BCIs in predicting which motor action was imagined by a subject. Our movement prediction algorithm uses Sequential Backward Selection technique to jointly choose temporal and spectral features and a radial basis function neural network for the classification. The method shows an average performance increase of 3.50% compared to state-of-the-art benchmark algorithms. Using two popular public datasets our algorithm reaches 90.08% accuracy (compared to an average benchmark of 79.99%) on the first dataset and 88.74% (average benchmark: 82.01%) on the second dataset. Given the high variability within- and across-subjects in EEG-based action decoding, we suggest that using features from multiple modalities along with neural network classification protocol is likely to increase the performance of BCIs across various tasks.

## Introduction

Brain-Computer Interfaces (BCIs) act as a link between neural activity and machine operations. The BCI extracts data from electrodes or sensors acquiring neural signals and translates those data into digital code (Bulárka and Gontean, 2016). Applications of BCI include those focused on improved health outcomes (i.e., rehabilitation of impaired motor function; Courtine et al., 2013), restoration of sensory functions (Hochberg et al., 2012), interpreting thoughts from individuals who cannot otherwise communicate them (Cerf et al., 2010), enhanced control of devices (i.e., operating heavy machinery, flying drones, or driving; Chiuzbaian et al., 2019, Nader et al., 2021), or recreational uses (i.e., gaming; Cerf and Garcia-Garcia, 2017). Invasive BCIs, such as ones built on single-neuron recordings, have recently shown high accuracy in interpreting human/animal intentions, actions, and imagery (Cerf et al., 2010; Hochberg et al., 2012). Non-invasive tools such as ones using electroencephalography (EEG) data have demonstrated high performance in interpreting thoughts and actions. For example, interpreting imagined motor action–a commonly used task for evaluating BCIs–has shown decoding accuracies ranging between 70 and 85% in recent works (Gordleeva et al., 2017).

Notably, BCIs based on motor imagination (MI) tasks, where subjects imagine an action (i.e., clenching of the fist) and the BCI aims to identify the action imagined, have shown remarkable improvement in recent years. In a typical MI task, the BCI derives neural signatures (i.e., power changes in the alpha and beta rhythms extracted from sensory-motor regions) that accurately predict the action intent following a training period. Given that non-invasive signals generated by EEG are often contaminated by artifacts derived from eye movement or muscle movement, a typical EEG-based BCI requires larger training data and isolated trials to increase the action identification accuracy. The repeated trials enable the averaging of the event-related signals and the extraction of a synchronized clean input. Variance across individual subjects, electrode montages, experimental sessions, and trial types add difficulty to the interpretation of the signals.

Given the challenges in EEG-based BCI development using noisy inputs, numerous methods have been proposed to improve the decoders performance (Lebedev and Nicolelis, 2006; Lotte et al., 2007; Prashant et al., 2015; Abiri et al., 2019; Andersen et al., 2019). The suggested methods often focus on the isolation of temporal or spectral components in the signal. Algorithms based on spectral feature selection are more prominent in the BCI arsenal since the time courses of event-related synchronization (or de-synchronization) vary heavily among subjects during motor tasks (Hochberg et al., 2012; Andersen et al., 2019).

Within the feature selection BCIs signal toolkit arsenal, common spatial patterns (CSPs) algorithms are dominant (Bhatti et al., 2019). These algorithms seek to find an optimal spatial filter that distinguishes one brain state from another. In EEG, the performance of CSPs is highly sensitive to the choice of frequency bands, making the decision on which filter to use heavily dependent on the recording configuration. To afford some generalization, variants of CSP were proposed as ways to improve the signal processing. Those variants often use narrower frequency bands (termed: sub-band CSP; SBCSP; Novi et al., 2007) and Filter Banks (FBCSP; Ang et al., 2008) and show increased performance for action decoding, yet are still scarce.

In addition to the extended frequency bands and filters improvements, recent attempts to include temporal signals in BCIs emerged in the form of Temporally Constrained Group Spatial Pattern (TSGSP) algorithms (Zhang et al., 2018). TSGSP optimally select the CSP features by considering different temporal windows for signal extraction derived from multi-task learnings. That is, instead of collapsing all the trials within one MI class (i.e., all left-hand movement trials) various MI tasks are combined to suggest the ideal CSP for a specific individual subject. The TSGSP algorithms use Support Vector Machines (SVM) for the classification of new trials to their corresponding action class. This inclusion of temporal data was recently shown to improve the performance of CSP-based BCIs (Sakhavi et al., 2018; Zhang et al., 2018; Deng et al., 2021).

Neural network based classifiers that frequently show superiority in data-rich non-linear clustering tasks such as MI decoding were recently suggested as a potential improvement for the CSP algorithms (Bhatti et al., 2019). Specifically, the usage of Sequential Backward Floating Selection method along with a radial basis function neural network (RBFNN) for optimal CSP features selection was suggested as a potential superior algorithm for BCIs (Bhatti et al., 2019).

Here we implement and test a combination of the suggested improvements for MI decoding and show the tuning curves of key parameters driving the performance increase. Namely, we introduce a number of additions to the BCI motor classification algorithms arsenal. First, we incorporate both temporal and spectral features in the MI BCI. Second, we use sub-bands rather than typical frequency bands for the BCI inputs. Third, we combine the successful Sequential Backward Selection (SBS) method with CSP features for the temporal-spectral feature selection. Fourth, we separate the feature selection process from the following feature classification process. Finally, we incorporate the suggested RBFNN (rather than SVM) in the motor classification. We demonstrate the effectiveness of our method using popular public datasets and compare our performance to the current state-of-the-art BCI benchmark algorithms.

This work contributes to the BCI literature by showing that the combination of a SBS and temporal-spectral EEG signals with RBFNN significantly outperforms other methods. This is the first work to test the combination of all previously suggested improvements to existing algorithms in a single implementation (see Sakhavi et al., 2018; Zhang et al., 2018; Deng et al., 2021; for discussions of the improvements implemented here).

## Materials and methods

### Data

Two popular BCI datasets were used for the algorithm testing:

#### Dataset 1

Brain-computer interface competition IV, dataset 2a, which contains 22-channel EEG data recorded from 9 healthy subjects (A01–A09) participating in different MI tasks. In each task, subjects were asked to imagine movement of the left hand, right hand, feet, and tongue. The experiment consisted of two sessions. In each session, there were 72 trials for each of the four classes of movement. The EEG signals were sampled at 250 Hz and bandpass filtered between 0.5 and 100 Hz with a 50 Hz notch filter. We used the data from the left- and right-hand imagery tasks alone to align with the second dataset and some of the benchmark algorithms that focused solely on those movement classes.

#### Dataset 2

Brain-computer interface competition IV, dataset 2b, which contains 3-channel EEG data recorded from 9 different subjects (B01–B09) participating in two MI tasks. The experimental protocol was nearly identical to dataset 1 other than the fact that subjects only imagined movements of the left-hand and right-hand, and that instead of two sessions there were five session. For each subject, separate training and testing sets were available. The EEG signals were sampled at 250 Hz and bandpass filtered between 0.5 and 100 Hz with a 50 Hz notch filter.

See Leeb et al. (2008) for additional details on the two datasets.

### Feature extraction

#### Pre-processing

Raw EEG signals were filtered between 4 and 40 Hz with fifth-order Butterworth filter. For each trial, we used samples between 500 and 4,500 ms from the trial onset in the analyses. The first 500 ms were excluded, in alignment with the instructions of the BCI IV competition winners, because of response times deviations across trials.

#### Feature selection

The neural signals were divided to five overlapping 2-s windows with a step size of 500 ms. This ensured temporal generalizability within a trial. Following, the data were filtered along 17 overlapping frequency bands ranging from 4 to 40 Hz with a 2 Hz step. Finally, a common spatial filter (Bhatti et al., 2019) was identified such that it maximized the variance within a single class (i.e., across all left-hand trials) and minimized the variance across classes (i.e., between left-hand and right-hand trials).

The data for a single trial were represented as a matrix, *X* ∈ *R*^*N*⋅*T*^ (with *N* reflecting the number of channels, and *T* the time) whose normalized covariance matrix, *C*, is:


(1) $$C=\frac{XX^{T}}{trace\left(XX^{T}\right)}$$

Averaging across all trials within a class yielded a matrix, *C*_*t*_ (*t* indicating the class type).

The spatial covariance was calculated by averaging all covariance matrices:


(2) $$C_{c}=\bar{C_{left-hand}}+\bar{C_{right-hand}}$$

The *C*_*c*_ matrix was white transformed:


(3) $$C_{C}=U_{C}\lambda_{C}U_{C}^{T}$$

with *U*_*C*_ the eigenvector matrix and λ_*C*_ the eigenvectors.

Defining *P* as:


(4) $$P=\sqrt{\lambda_{C}^{-1}}U_{C}^{T}$$

the individual class matrices were transformed to:


(5) $$S_{left-hand}=P\bar{C_{left-hand}}P^{T}$$

(6) $$S_{right-hand}=P\bar{C_{right-hand}}P^{T}$$

such that the *S*_*t*_ matrices have the same eigenvectors.

Given that *S*_*t*_ could be represented as *B*λ_*t*_*B^T^* with *B* the eigenvectors matrix and λ_*t*_ the eigenvalues:


(7) $$S_{t}=B\lambda_{t}B^{T}$$

the projection matrix, *W*, was derived:


(8) $$W=B^{T}P$$

Thus, the EEG data were projected to a matrix, *Z*:


(9) $$Z=W^{T}X$$

where the columns of *Z* corresponded to the data’s spatial source distribution vectors. The vectors maximized the variance across classes and corresponded to the maximum eigenvalues (λ_*left–hand*_ and λ_*right*−*hand*_).

Finally, the classification features were represented by:


(10) $$f_{p}=\log\left(\frac{var\left(Z_{p}\right)}{\sum_{i=1}^{n}var\left(Z_{i}\right)}\right)$$

where *Z*_*p*_ are the CSPs (*p* = *1*..*N*).

A subset of *Z* (first and last *m* rows) were used in further analyses.

An SBS (Pasyuk et al., 2019) was used to reduce the initial 85-feature set (17 frequency bands × 5 time-windows) from each individual trial. According to the SBS criteria, in every iteration of the algorithm the feature yielding the lowest accuracy was discarded. That is, if the initial performance with all 85 features was, say, 87%, the performance using 84 features was computed next, leaving one feature out in each iteration (*f*_*1*_ = 78%, *f*_*2*_ = 82%, *f*_*3*_ = 77%, …). Comparing all 85 leave-one-out iterations, the feature whose contribution to the performance was lowest (i.e., one without whom the performance drops least; *f*_*2*_ in the particular example) was discarded. Following, the performance of the remaining 84 features was set as the anchor performance and the evaluation was repeated with 83 features. Each run led to a drop of a single feature. The optimal performance across all 3,655 iterations (85+84+…) was regarded the network’s accuracy, with the feature set yielding the highest performance being the preferred set.

### Neural network

An RBFNN was used for the classification. The network consisted of two layers: an input layer and a hidden layer. The output of the hidden layer was summed proportionally to the input features to yield the output classification. Formally, this is represented as:


(11) $$F\left(x\right)=\sum_{i=1}^{k}{\text{w}}_{i}{\text{f}}_{i}\left(x,{\text{c}}_{i}\right)+b$$

where w*_i_* are the weights, f*_i_* the Gaussian radial basis functions, c*_i_* the center values of the Gaussian radial function, *b* the bias, and *k* the number of neurons in the hidden layer.

With f*_i_* formally computed as:


(12) $$f_{i}\left(x,{\text{c}}_{i}\right)=e^{\left(\frac{-{||{\mathrm{x-c}}_{i}||}^{2}}{2\sigma_{i}^{2}}\right)}$$

where σ_*i*_ is the standard deviation.

In each iteration of the RBFNN implementation the extracted input features are scaled and used to train the network, followed by a testing. The network was implemented using Matlab’s *newrbe* function default hyperparameters, with the spread of the radial basis functions set to 16.

### Implementation

The implementation of the method–pre-processing, feature selection, and neural network classification are available online at https://www.morancerf.com/publications.

#### Analyses

We compared our algorithm’s performance to that of all state-of-the-art methods which: (a) were published in the last 5 years, (b) used the same datasets as ours, and (c) were implemented on both the left- and right-hand MI data. We used one implementation of each method to avoid focusing on coding variations but rather on conceptual differences in the protocol. Altogether, 38 methods were compared to our algorithms, and 19 were not included in our analyses because they did not satisfy the inclusion criteria (namely, those algorithms used different movement classes outside of the ones we tested).

For dataset 1 we compared our performance to the following methods (see results in Table 1):

**TABLE 1:** Performance comparison for dataset 1, sorted by accuracy.

Method	Year	A01	A02	A03	A04	A05	A06	A07	A08	A09	Mean ± std
SBS-FBCSP	2022	80.00	72.76	83.79	70.42	73.10	68.97	75.17	77.93	77.59	75.52 ± 4.76
DNN	2016	86.81	66.70	95.83	76.39	57.64	68.06	75.00	93.75	77.08	77.47 ± 12.72
KPCA CILK	2016	88.89	59.03	90.28	78.47	62.50	75.00	72.92	93.06	87.50	78.63 ± 12.34
WOLA-CSP	2018	86.81	63.19	94.44	68.75	56.25	69.44	78.47	97.91	93.75	78.78 ± 15.15
MEMDBF-CSP-LDA	2019	90.78	57.75	97.08	70.69	61.48	70.37	72.14	97.76	94.62	79.19 ± 15.85
JSTFD-LDA	2020	86.40	55.90	96.30	73.10	89.50	58.20	76.10	93.80	86.60	79.54 ± 14.78
nCSP-TSLR	2019	89.23	76.15	90.60	71.38	59.82	63.26	91.70	89.18	85.26	79.62 ± 12.36
W-CNN	2019	76.67	72.00	90.00	73.33	83.33	80.00	82.67	80.00	80.00	79.78 ± 5.45
SS-MEMDBF	2018	91.49	60.56	94.16	76.16	58.52	68.52	78.57	97.01	93.85	79.87 ± 15.01
CSP-Wavelet + LOG	2020	93.06	61.81	95.83	72.92	58.33	68.06	81.25	95.14	93.06	79.94 ± 15.06
SW-LSR	2021	86.81	64.58	95.83	67.36	68.06	67.36	80.56	97.22	92.36	80.02 ± 13.45
EEGnet	2016	71.43	78.51	100	64.28	71.43	78.57	71.43	92.86	100	80.95 ± 13.37
R-MDRM	2019	91.61	63.28	97.20	72.91	64.08	69.71	81.25	96.52	92.30	80.98 ± 13.86
SR-MDRM	2019	90.21	63.28	96.55	76.38	65.49	69.01	81.94	95.14	93.01	81.22 ± 13.19
TSGSP	2018	87.00	64.70	93.80	74.30	90.40	63.90	91.40	95.80	81.30	82.51 ± 12.24
DCR-MEMD	2021	89.79	94.18	78.92	94.01	71.32	86.71	89.36	82.11	86.18	85.84 ± 7.40
*Ours*	*2022*	*93.45*	*84.83*	*95.52*	*88.33*	*86.55*	*83.10*	*88.97*	*95.52*	*94.48*	*90.08* ± 4.78

(1) Deep Neural Network (DNN) (Kumar et al., 2016)
(2) Kernel Principal Component Analysis using Conformal-Isometric Linearizing Kernel (KPCA-CILK) (Sadatnejad and Ghidary, 2016)
(3) Weighted Overlap Add Common Spatial Patterns (WOLA-CSP) (Belwafi et al., 2018)
(4) Multivariate Empirical Mode Decomposition Based Filtering-Common Spatial Pattern-Linear Discriminant Analysis (MEMDBF-CSP-LDA) (Gaur et al., 2019)
(5) Joint Spatio-temporal Filter Design Linear Discriminant Analysis (JSTFD-LDA) (Jiang et al., 2020)
(6) Normalized Common Spatial Pattern Tangent Space Logistic Regression (nCSP-TSLR) (Olias et al., 2019)
(7) Wavelet Convolutional Neural Network (W-CNN) (Xu et al., 2018)
(8) Subject Specific Multivariate Empirical Mode Decomposition Based Filtering (SS-MEMDBF) (Gaur et al., 2018)
(9) Common Spatial Pattern-Filter Bank-Log (CSP-FB-LOG) (Zhang S. et al., 2020)
(10) Sliding Window-Longest Consecutive Repetition (SW-LSR) (Gaur et al., 2021)
(11) EEG Network (EEGnet) (Lawhern et al., 2018)
(12) Regularized Minimum Distance to Riemannian Mean (R-MDRM) (Singh et al., 2019)
(13) Spatial Regularized Minimum Distance to Riemannian Mean (SR-MDRM) (Singh et al., 2019)
(14) Temporally Constrained Sparse Group Spatial Patterns (TSGSP) (Zhang et al., 2018)
(15) Dynamic Channel Relevance-Multivariate Empirical Mode Decomposition (DCR-MEMD) (Song and Sepulveda, 2018)

For dataset 2 we compared our results to the following methods (see results in Table 2):

**TABLE 2:** Performance comparison for dataset 1, sorted by accuracy.

Method	Year	B01	B02	B03	B04	B05	B06	B07	B08	B09	Mean ± std
SBS-FBCSP	2022	70.14	60.29	62.50	89.19	82.43	73.61	66.67	74.34	79.86	73.23 ± 9.49
RSMM	2016	72.50	56.43	55.63	97.19	88.44	78.75	77.50	91.88	83.44	77.97 ± 14.56
DLAV	2019	76.10	67.30	71.80	95.40	82.30	82.10	77.50	75.30	75.90	78.19 ± 7.95
SGRM	2019	76.30	56.00	49.20	98.20	91.10	74.80	88.30	85.40	84.90	78.24 ± 16.26
UDFS	2019	76.09	58.64	53.45	99.38	83.83	76.96	83.15	90.66	83.48	78.40 ± 14.53
SSD-SE-CNN	2021	78.50	67.90	68.30	96.50	81.40	85.70	76.90	79.30	79.60	79.34 ± 8.65
WaSF ConvNet	2019	73.80	64.20	85.70	96.20	85.20	68.50	88.30	90.10	81.50	81.50 ± 10.58
NCFS	2020	79.25	63.48	56.65	99.28	88.67	79.96	88.76	92.66	84.95	81.52 ± 13.72
CSP-FB-LOG	2020	88.75	52.50	48.75	98.75	88.75	90.00	90.00	92.50	83.75	81.53 ± 17.98
MAAN	2021	82.81	60.36	59.06	97.50	91.88	86.38	84.06	93.44	86.88	82.49 ± 13.73
MTPP-EEGNet	2020	78.75	66.43	67.50	95.00	94.38	84.38	85.31	92.19	81.56	82.83 ± 10.61
DJDA	2021	83.44	58.57	59.06	98.13	96.56	84.38	86.25	92.81	87.81	83.00 ± 14.64
SHNN	2022	83.33	61.76	58.33	97.30	91.89	88.89	86.11	92.11	91.67	83.49 ± 13.89
TSLDA	2019	76.30	68.90	86.40	94.20	88.10	72.30	89.20	92.80	87.30	83.94 ± 9.13
DRDA	2021	81.37	62.86	63.63	95.94	93.56	88.19	85.00	95.25	90.00	83.98 ± 12.67
RF-DFFS	2016	73.24	67.48	63.01	97.40	95.49	86.66	84.68	95.93	92.61	84.06 ± 13.06
FDBN	2016	81.00	65.00	66.00	98.00	93.00	88.00	82.00	94.00	91.00	84.22 ± 11.94
TSGSP	2018	84.00	62.60	56.30	99.40	94.80	83.80	94.10	93.30	90.10	84.27 ± 15.01
MMCNN	2020	84.90	70.40	75.50	96.30	92.40	86.30	87.60	84.20	81.80	84.38 ± 7.92
WPD-STDF	2019	69.50	64.00	86.50	96.00	94.00	87.00	83.00	95.50	92.00	85.28 ± 11.47
CD-CNN	2021	79.69	60.71	82.19	96.87	94.37	89.37	82.19	93.75	90.00	85.46 ± 11.08
FBRTS	2022	82.40	75.20	86.90	95.20	89.70	80.20	90.50	91.20	91.10	86.93 ± 6.40
*Ours*	*2022*	*90.28*	*75.00*	*73.61*	*100*	*97.30*	*90.28*	*84.03*	*92.11*	*95.83*	*88.72* ± *9.40*
Li M.-A. et al., 2019	2019	–	–	–	–	–	–	–	–	–	96.48
Li M. et al., 2021	2021	–	–	–	–	–	–	–	–	–	97.03

Two of the algorithms that were excluded from our benchmark comparisons are shown in the table in gray. These algorithms were excluded since they could only be implemented on the second dataset. However, since they showed higher performance than ours on we listed them here to highlight their potential superiority (no individual subject data were available for these works, hence we only show the overall average performance).

(1) Robust Support Matrix Machine (RSMM) (Zheng et al., 2018)
(2) Deep Learning with Variational Autoencoder (DLVA) (Dai et al., 2019)
(3) Sparse Group Representation Model (SGRM) (Jiao et al., 2018)
(4) Unsupervised Discriminative Feature Selection (UDFS) (Al Shiam et al., 2019)
(5) Sparse Spectro-temporal Decomposition Squeeze-and-Excitation Convolutional Neural Network (SSD-SE-CNN) (Sun et al., 2020)
(6) Wavelet Spatial Filter Convolution Network (WaSF ConvNet) (Dy et al., 2019; Fang et al., 2022)
(7) Neighborhood Component analysis based Feature Selection (NCFS) (Molla et al., 2020)
(8) Common Spatial Pattern-Wavelet-Log (CSP-Wavelet-LOG) (Zhang S. et al., 2020)
(9) Multi-Attention Adaptation Network (MAAN) (Chen et al., 2021)
(10) Multilayer Temporal Pyramid Pooling EEG Network (MTPP-EEGNet) (Ha and Jeong, 2020)
(11) Dynamic Joint Domain Adaptation (DJDA) (Hong et al., 2021)
(12) SincNet-based Hybrid Neural Network (SHNN) (Liu et al., 2022)
(13) Tangent Space Linear Discriminant Analysis (TSLDA) (Ai et al., 2019; Fang et al., 2022)
(14) Deep Representation-based Domain Adaptation (DRDA) (Zhao et al., 2020)
(15) Random Forest Dynamic Frequency Feature Selection (RF-DFFS) (Luo et al., 2016)
(16) Frequential Deep Belief Network (FDBN) (Lu et al., 2016)
(17) Temporally constrained Sparse Group Spatial Patterns (TSGSP) (Zhang et al., 2018)
(18) Multi-branch Multi-scale Convolutional Neural Network (MMCNN) (Jia et al., 2020)
(19) Wavelet Package Decomposition Spatio-Temporal Discrepancy Feature (WPD-STDF) (Luo et al., 2019)
(20) Central Distance Loss Convolutional Neural Network (CD-CNN) (Yang et al., 2021)
(21) Filter Banks and Riemannian Tangent Space (FBRTS) (Fang et al., 2022)

Additionally, we implemented a version of the Sequential Backward Selection Filter Bank Common Spatial Patterns (SBS-FBCSP) algorithm, which is an adaptation of the Sub-Band Common Spatial Patterns with Sequential Backward Floating Selection (SBCSP-SBFS) proposed by Bhatti et al. (2019). The original SBCSP-SBFS algorithm did not use temporal features and was limited to 12 overlapping frequency bands (4–30 Hz). Conceptually, the SBS-FBCSP algorithm resembled our method in that it, too, used sub-bands and CSPs feature selection. SBS-FBCSP differed from our method in that it used the full trial as temporal dimension.

For dataset 1, we varied the parameter *m* from 1 to 7 (with *2m* options yielding up to 14 features in each trial) since the parameter selection impacts the performance. To calculate the accuracy, we used 5-fold cross validation with all the trials from the first dataset (combining the first and second sessions onto one data set).

For dataset 2 we varied *m* from 1 to 3 yielding up to six features. To calculate the accuracy, we combined all sessions data in random order and used 80% of the trials for training and the remaining 20% for testing sessions as training set and the remaining two for testing, with five-fold cross validation (see Luo et al., 2016).

The excluded methods were:

(1) Distance Preservation to Local Mean (Davoudi et al., 2017)

(2) Neighborhood Rough Set Classifier (Udhaya Kumar and Hannah Inbarani, 2017)

(3) Channel-wise Convolution with Channel Mixing (Sakhavi et al., 2018)

(4) Gated Recurrent Unit Recurrent Neural Network Long-Short Term Memory-Recurrent Neural Network (Luo et al., 2018)

(5) Deep Recurrent Spatial-Temporal Neural Network (Ko et al., 2018)

(6) Long-Short Term Memory network (Wang et al., 2018)

(7) Dempster-Shafer Theory (Razi et al., 2019)

(8) Densely Feature Fusion convolutional neural Network (Li D. et al., 2019)

(9) Convolutional Neural Network Long-term Short-term Memory Network (Zhang R. et al., 2019)

(10) Multi-branch 3D Convolutional Neural Network (Zhao et al., 2019)

(11) Channel-Projection Mixed-scale convolutional neural Network (Li Y. et al., 2019)

(12) Convolutional Recurrent Attention Model (Zhang D. et al., 2019)

(13) Weight-based Feature Fusion Convolutional Neural Network (Amin et al., 2019)

(14) Multi-Scale Fusion Convolution Neural Network (Li D. et al., 2020)

(15) Multiple Kernel Stein Spatial Patterns (Galindo-Noreña et al., 2020)

(16) Graph-based Convolutional Recurrent Attention Model (Zhang D. et al., 2020)

(17) Temporal-Spatial Convolutional Neural Network (Chen et al., 2020)

(18) Temporal-Spectral-based Squeeze-and-Excitation Feature Fusion Network (Li Y. et al., 2021)

(19) Shallow Convolution Neural Network and Bidirectional Long-Short Term Memory (Lian et al., 2021)

(20) Temporal Convolutional Networks-Fusion (Musallam et al., 2021)

(21) EEG-Inception-Temporal Network (Salami et al., 2022)

## Results

### Performance

Our algorithm, which we term Sequential Backward Selection with Temporal Filter Bank Common Spatial Patterns (SBS-TFBCSP), significantly outperformed the average performance (79.99% ± 2.23; mean ± std) of all other algorithms. By 12.61% (*T*(8) = 5.057, *p* < 0.001; *t*-test; Table 1) and outperformed each of those algorithms individually. The algorithm outperformed the contender leading algorithm (DCR-MEMD) by 4.94%, yet this was not significant (*T*(8) = 1.322, *p* = 0.223, *t*-test). While conceptually similar, the SBS-FBCSP yielded the lowest score among the methods compared.

Using the second dataset, our algorithm significantly outperformed the average (82.01% ± 3.25) of all other algorithms by 8.18% (*T*(8) = 5.697, *p* < 0.001; *t*-test) and each of those algorithms individually (Table 2). Comparing our algorithm’s performance to the leading state-of-the-art contender algorithm (FBRTS), we see a non-significant 2.06% increase in performance favoring our method (*T*(8) = 0.707, *p* = 0.499, *t*-test). The SBS-FBCSP again yielded the lowest performance among the methods compared.

**FIGURE 1:**
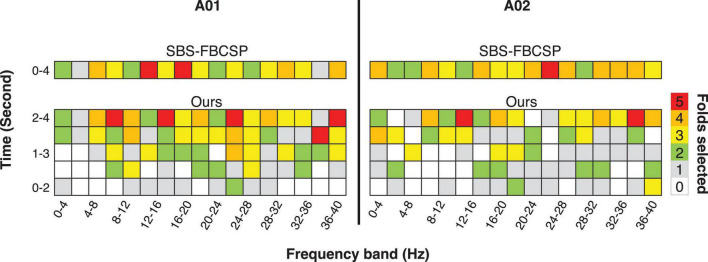
Illustration of the results of the 5-fold feature selection comparing a contender algorithm (SBS-FBCSP) and our algorithm, which namely differ in the breakdown of temporal features. The colors (taken from subject A01, A02; chosen arbitrarily) denote the number of times a feature-subset was selected during the 5-fold cross validation.

Noting that the performance of SBS-FBCSP is lower across datasets while the key difference between our algorithm and the SBS-FBCSP is the features selected, we suggest that the inclusion of temporal features in the CSPs is likely driving the performance increase (Figure 1). The expansion of the frequency range implemented in our algorithm increases the feature selection granularity, and in turn the performance. As an intuition for the advantage of the method with respect to the feature selection, we show examples (subjects A01, A02; chosen arbitrarily; Figure 1) where the feature-subsets selected by the algorithms are highlighted. In both subjects, a larger proportion of the selected features were drawn from the last 2 s (which SBS-FBCSP would ignore since it averages across the entire 4-s window). Additionally, a number of the selected features were drawn from frequency bands above 30 Hz which would be excluded in the standard SBCSP-SBFS implementations (Bhatti et al., 2019) because they correspond to frequencies not typically associated with MI.

To further investigate the difference between our work and similar methods we highlight two additional algorithms that share various features with ours. The Sparse Filter Bank Common Spatial Pattern (SFBCSP) and the Multiple Windows SFBCSP (SFBCSP-MT) both used a feature selection to choose the CSP features from multiple filter banks (SFBCSP) and 5 (dataset 1) or 6 (dataset 2) time windows (SFBCSP-MT). However, the contender algorithms did not use SBS. Our method outperformed the SFBCSP contender algorithm among eight of the nine subjects using both dataset 1 and 2, and among seven (dataset 1) and eight (dataset 2) of the nine subjects with the SFBCSP-MT contender algorithm (Tables 3, 4).

**TABLE 3:** Performance comparisons of our method and two similar ones, for dataset 1.

Measurement	A01	A02	A03	A04	A05	A06	A07	A08	A09	Mean ± std
SFBCSP	82.80	59.80	92.00	68.00	81.03	59.30	89.10	92.80	70.20	77.30 ± 13.30
SFBCSP (MT)	84.10	62.90	92.90	71.60	86.90	61.20	89.80	94.30	80.90	80.50 ± 12.50
*Ours*	*93.45*	*84.83*	*95.52*	*88.33*	*86.55*	*83.10*	*88.97*	*95.52*	*94.48*	*90.08* ± 4.78

Performance metrics for the algorithms were taken from Zhang et al. (2018).

**TABLE 4:** Performance comparisons of our method and two similar ones, for dataset 2.

Measurement	B01	B02	B03	B04	B05	B06	B07	B08	B09	Mean ± std
SFBCSP	79.10	59.00	53.10	98.90	91.50	81.30	90.80	88.90	85.40	80.90 ± 15.30
SFBCSP (MT)	81.80	60.30	54.00	99.10	92.60	82.00	91.80	91.10	87.30	82.20 ± 15.30
*Ours*	*90.28*	*75.00*	*73.61*	*100*	*97.30*	*90.28*	*84.03*	*92.11*	*95.83*	*88.72* ± *9.40*

Performance metrics for the algorithms were taken from Zhang et al. (2018).

Taken together, our results suggest that the performance increase is driven by broader choice of inputs, and the feature selection process.

#### Results of other performance measure

To further evaluate the performance of our algorithm we used additional standard accuracy metrics. Namely, we estimated the Positive Precision Value (PPV, TPTP+FP), Negative Precision Value (NPV, TNTN+FN), sensitivity (True Positive Rate, TPTP+FN), specificity (True Negative Rate, TNTN+FP), and Kappa value (Po−Pe1−Pe), where TP represents the number of testing samples whose real value aligned with the model prediction (True Positive), TN represents the number of testing samples whose real value and model predicted values were both negative (True Negative), FP represents the number of testing samples whose real value is negative while their model predicted value is positive (False Positive), and FN represents the number of testing samples whose real value is positive while their model predicted value is negative (False Negative). *P*_*o*_ is the proportion of observed agreement, and *P*_*e*_ probability that the agreement is at chance. In both dataset 1 (Table 5) and dataset 2 (Table 6) our algorithm proved superior compared to the SBS-FBCSP using those metrics.

**TABLE 5:** Performance comparison between SBS-FBCSP and our method, using dataset 1.

Measurement	A01	A02	A03	A04	A05	A06	A07	A08	A09	Mean ± std
SBS-FBCSP										
PPV	81.10	74.57	85.91	70.78	75.19	72.63	75.00	80.28	78.65	77.12 ± 4.75
NPV	80.04	72.64	82.97	71.93	71.46	67.41	76.85	77.52	77.41	75.36 ± 4.85
TPR	79.31	71.03	82.07	71.67	68.97	62.07	77.24	77.24	76.55	74.02 ± 6.15
TNR	80.69	75.17	85.52	69.17	77.24	75.86	73.10	78.62	78.62	77.11 ± 4.64
Kappa	60.00	46.21	67.59	40.83	46.21	37.93	50.34	55.86	55.17	51.13 ± 9.47
*Ours*										
PPV	94.58	85.77	94.57	88.65	88.48	82.28	90.51	94.75	92.40	90.22 ± 4.35
NPV	92.89	85.48	96.62	88.39	87.62	86.56	88.10	96.56	97.35	91.06 ± 4.79
TPR	92.41	84.14	96.55	88.33	85.52	86.21	87.59	96.55	97.24	90.50 ± 5.23
TNR	94.48	85.52	94.48	88.33	87.59	80.00	90.34	94.48	91.72	89.66 ± 4.89
Kappa	86.90	69.66	91.03	76.67	73.10	66.21	77.93	91.03	88.97	80.17 ± 9.56

**TABLE 6:** Performance comparison between SBS-FBCSP and our method, using dataset 2.

Measurement	B01	B02	B03	B04	B05	B06	B07	B08	B09	Mean ± std
SBS-FBCSP										
PPV	65.93	61.67	62.50	87.18	83.33	72.97	64.29	74.03	77.22	72.12 ± 9.25
NPV	77.36	59.21	62.50	91.43	81.58	74.29	70.00	74.67	83.08	74.90 ± 10.09
TPR	83.33	54.41	62.50	91.89	81.08	75.00	75.00	75.00	84.72	75.88 ± 11.51
TNR	56.94	66.18	62.50	86.49	83.78	72.22	58.33	73.68	75.00	70.57 ± 10.49
Kappa	40.28	20.59	25.00	78.38	64.86	47.22	33.33	48.68	59.72	46.45 ± 18.97
*Ours*										
PPV	86.25	73.61	75.00	100	97.30	92.65	78.82	92.00	95.83	87.94 ± 9.95
NPV	95.31	76.56	72.37	100	97.30	88.16	91.53	90.91	95.83	89.77 ± 9.44
TPR	95.83	77.94	70.83	100	97.30	87.50	93.06	90.79	95.83	89.90 ± 9.68
TNR	84.72	72.06	76.39	100	97.30	93.06	75.00	92.11	95.83	87.39 ± 10.60
Kappa	80.56	50.00	47.22	100	94.59	80.56	68.06	82.89	91.67	77.28 ± 18.74

Specifically, with respect to dataset 1, our algorithm significantly outperformed the PPV of the SBS-FBSP (77.12% ± 4.75) by 16.99% (*T*(8) = 13.653, *p* < 10^–7^; *t*-test), the NPV of the SBS-FBSP (75.36% ± 4.85) by 20.83% (*T*(8) = 14.704, *p* < 10^–7^; *t*-test), the TPR of the SBS-FBSP (74.02% ± 6.15) by 22.26% (*T*(8) = 11.469, *p* < 10^–6^; *t*-test), the TNR of the SBS-FBSP (77.11% ± 4.64) by 16.28% (*T*(8) = 8.125, *p* < 10^–5^; *t*-test), and the Kappa of the SBS-FBSP (51.13% ± 9.47) by 56.80% (*T*(8) = 18.318, *p* < 10^–8^; *t*-test).

In dataset 2, our algorithm again significantly outperformed the PPV of the SBS-FBSP (72.12% ± 9.25) by 21.94% (*T*(8) = 14.33, *p* < 10^–7^; *t*-test), the NPV of the SBS-FBSP (74.90% ± 10.09) by 19.85% (*T*(8) = 10.952, *p* < 10^–6^; *t*-test), the TPR of the SBS-FBSP (75.88% ± 11.51) by 18.48% (*T*(8) = 8.514, *p* < 10^–5^; *t*-test), the TNR of the SBS-FBSP (70.57% ± 10.49) by 23.83% (*T*(8) = 8.169, *p* < 10^–5^; *t*-test), the Kappa of the SBS-FBSP (46.45% ± 18.97) by 66.37% (*T*(8) = 15.479, *p* < 10^–7^; *t*-test).

### Parameters sensitivity

Given that the performance of our proposed method heavily depends on the selection of the *m* parameter we tested the robustness of our results by enumerating over all *m* values possible in dataset 1 (Table 7) and dataset 2 (Table 8). While, indeed, the choice of *m* impacts the algorithm performance across subjects, the average difference in performance for dataset 1 was 2.23% ± 0.85 and average difference in performance for dataset 2 of 0.99% ± 0.67 (with the highest drop in performance yielding 83.94% accuracy). The lowest performance was aligned with the accuracy of the DCR-MEMD algorithm, but better than all other methods. The highest performance drop yielded an accuracy of 86.79%. which was on par with the FBRTS method but better than all other methods. Combined, these results suggest that the method is robust to perturbations of its single free parameter and maintains its efficiency irrespective of the parameter choice.

**TABLE 7:** Performance comparison of different values of *m* for our method, using dataset 1.

*m*	A01	A02	A03	A04	A05	A06	A07	A08	A09
1	84.48	80.69	88.28	83.75	82.41	77.24	82.41	86.56	89.66
2	88.97	81.38	92.41	84.58	84.83	80.69	85.52	86.90	93.10
3	89.31	83.79	94.83	84.58	84.83	82.76	85.52	90.69	93.79
4	91.38	84.83	94.83	85.00	85.52	83.10	85.17	93.10	94.14
5	93.45	81.38	95.52	88.33	84.14	81.38	87.24	94.83	93.79
6	92.41	83.45	95.52	86.25	86.55	82.41	88.62	95.17	93.45
7	93.45	82.41	93.79	87.50	83.79	82.41	88.97	95.52	94.48
STD	3.21	1.51	2.59	1.70	1.32	2.02	2.27	3.85	1.62

**TABLE 8:** Performance comparison of different values of *m* for our method, using dataset 2.

*m*	B01	B02	B03	B04	B05	B06	B07	B08	B09
1	90.28	75.00	72.92	100	93.92	90.28	84.03	91.47	93.75
2	88.89	72.79	73.61	100	97.30	90.28	84.03	92.11	95.83
3	89.58	70.69	72.22	100	95.95	88.89	83.33	90.13	93.06
STD	0.70	2.16	0.70	0	1.70	0.80	0.40	1.01	1.44

Additionally, as our algorithm used temporal windows similar to those suggested in previous work (Zhang et al., 2018), yet the selection of number of windows in both ours and the previous work was arbitrary, we estimated the sensitivity of the algorithm to the selection of window sizes. We altered the number of temporal windows used from 4 to 6 to see the impact of this change on the accuracy. We used this range under the assumption that keeping the number of windows proportional to the number of frequency bands would align with existing works and the theoretical reasoning that they suggest for the bin sizes (Zhang et al., 2018). Testing the algorithm with varying window sizes shows that the range of perturbations yields a performance change of ±1.74%, proportional to the number of windows used. While manipulating the window size impacted the performance, the change was not significant. That is, the impact of ±1 window size usage had a marginal difference in performance (±1.29% on average for dataset 1, and ±0.87% for dataset 2). This non-significant change in performance along with the fact that a change from a single window (SBS-FBCSP) to 5 bins (ours) yields a notable difference suggest that there is a plateau in the performance increment after four bins.

#### Ablation study

**FIGURE 2:**
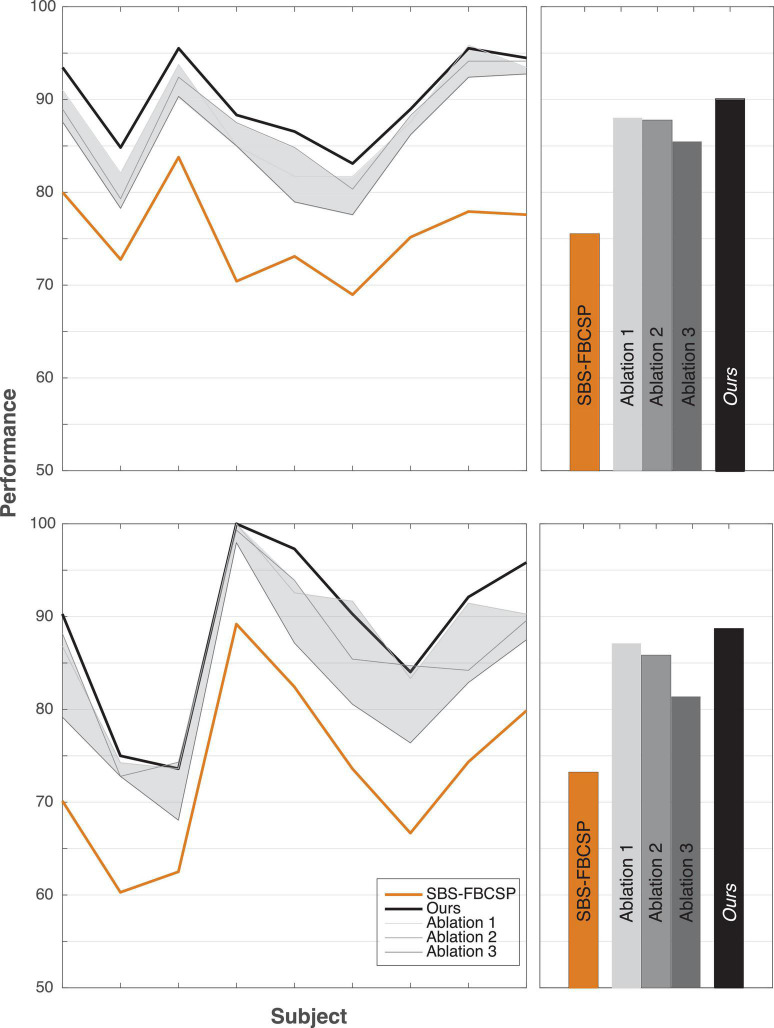
Ablation tests. Reducing the number of features as ablation tests for dataset 1 **(top row)** and dataset 2 **(bottom row)**. The shaded areas depict the range of performance for all nine subjects across all ablation tests, with the three tests showing the extremal performance highlighted individually as “Ablation #.” Right panels show the average performance across all subjects. “Ablation 1” corresponds to a test that included all five time windows (500–4,500 ms range, with 2 s windows size, and 500 ms step size) and 12 frequency bands (4–30 Hz range, with 4 Hz window size, and 2 Hz step size) for a total of 60 input features (12 bands × 5 time windows) reduced gradually to 10 features through the selection. “Ablation 2” corresponds to a test with three time windows (500–3,500 ms range, with 2 s windows size, and 500 ms step size) and 17 frequency bands (4–40 Hz range, with 4 Hz window size, and 2 Hz step size) for a total of 51 input features (17 bands × 3 time windows) reduced gradually to 10 features through the selection. “Ablation 3” corresponds to a test with three time windows (500–3,500 ms range, with 2 s windows size, and 500 ms step size) and 12 frequency bands (4–30 Hz range, with 4 Hz window size, and 2 Hz step size) for a total of 36 input features (12 bands × 3 time windows) reduced gradually to 10 features through the selection.

To further investigate the validity of the proposed algorithm we conducted a series of tests where we hindered the algorithm’s inputs and evaluated the performance change. As one key difference between our algorithm and existing ones is the inclusion of both temporal and spectral bands, we varied both input features. In a series of ablation studies, we decreased the range of spectral features from 17 (our algorithm) to 12 (as is done in contender algorithms) and the range of temporal features from five (our algorithm) to one (as is done in contender algorithms, namely SBS-FBCSP). Across all tests, the feature selection (Sequential Backward Selection) and the classifications parameters were held constant. Across all ablation tests, the performance drop ranged from −4.62% to −2.05% for dataset 1, and −7.33% to −1.61% for dataset 2. Our algorithm remained on par with the state-of-the-art benchmarks despite the drop in performance. The algorithm maintained its superiority for dataset 1, and ranked 15th (out of 21) for dataset 2 at its most hindered state, when the number of frequency bands used was lowest. That is, the selection of time windows and frequency bands that led to our algorithm’s performance seem to be mostly sensitive to the number of frequency bands used as inputs (Figure 2). Importantly, the drop in frequency bands to a lower number puts our algorithm in line with the contender ones, suggesting that some of the improvement is contingent on this input feature broadening.

Additionally, we replicated the accuracy metrics tests with the ablated inputs to evaluate the impact of the input on performance in an additional manner (Table 9). We attempted various implementations of the model with input features ranging from 12 to 17 frequency bands and 3–5 temporal windows. Our algorithm significantly outperformed a variety of contender algorithms with ablated input. Highlighting three of the ablation studies (“Ablation 1” with 60 input features, “Ablation 2” with 51 input features, and “Ablation 3” with 36 input features), our algorithm maintained its performance improvement. Specifically, for ablation test “1” our algorithm outperformed the non-ablated input by over 2% (*T*(8) = 4.143, *p* = 0.003; *t*-test) using dataset 1, and by over 1.5% using dataset 2 (*T*(8) = 2.024, *p* = 0.078; *t*-test). Similarly, in ablation test “2,” dataset 1 (*T*(8) = 3.869, *p* = 0.005; *t*-test), dataset 2 (*T*(8) = 2.883, *p* = 0.020; *t*-test) as well as in ablation test “3,” dataset 1 (*T*(8) = 7.051, *p* < 10^–4^; *t*-test) and dataset 2 (*T*(8) = 6.553, *p* < 10^–4^; *t*-test) the performance was consistency significantly higher for the non-ablated implementation.

**TABLE 9:** Details of three ablation tests using our algorithm in dataset 1 and 2.

Measurement	A01	A02	A03	A04	A05	A06	A07	A08	A09	Mean ± std	Significance [*T*(8) = (*T*,*p*, *t*-test)]
Ablation 1											
PPV	89.94	84.06	92.35	83.33	84.94	83.32	89.52	97.23	91.75	88.49 ± 4.88	2.033, 0.076
NPV	92.38	82.36	95.77	87.50	79.92	81.20	86.39	94.83	95.76	88.46 ± 6.43	3.232, **0.012**
TPR	92.41	80.69	95.86	88.33	77.93	80.00	85.52	94.48	95.86	87.90 ± 7.16	2.908, **0.020**
TNR	89.66	83.45	91.72	81.67	85.52	83.45	89.66	97.24	91.03	88.16 ± 5.02	1.394, 0.201
Kappa	82.07	64.14	87.59	70.00	63.45	63.45	75.17	91.72	86.90	76.05 ± 11.34	4.140, **0.003**
Ablation 2											
PPV	88.54	79.32	94.15	89.93	84.94	76.19	90.06	94.69	92.96	87.86 ± 6.51	2.235, 0.056
NPV	89.90	80.00	91.81	86.01	84.97	86.98	87.42	93.89	96.04	88.56 ± 4.91	4.034, **0.004**
TPR	89.66	80.00	91.03	85.00	84.83	88.97	86.21	93.79	95.86	88.37 ± 4.91	2.701, **0.027**
TNR	88.28	78.62	93.79	90.00	84.83	71.72	90.34	94.48	92.41	87.16 ± 7.59	2.021, 0.078
Kappa	77.93	58.62	84.83	75.00	69.66	60.69	76.55	88.28	88.28	75.54 ± 10.95	3.871, **0.005**
Ablation 3											
PPV	87.23	80.10	88.30	86.12	79.23	78.15	85.33	91.70	93.07	85.47 ± 5.36	4.870, **0.001**
NPV	88.94	77.16	92.92	84.55	80.72	78.15	88.37	93.82	92.59	86.36 ± 6.47	5.048, **10^–4^**
TPR	88.28	75.86	93.10	84.17	78.62	77.24	88.28	93.79	92.41	85.75 ± 7.07	4.792, **0.001**
TNR	86.90	80.69	87.59	85.83	79.31	77.93	84.14	91.03	93.10	85.17 ± 5.18	4.313, **0.003**
Kappa	75.17	56.55	80.69	70.00	57.93	55.17	72.41	84.83	85.52	70.92 ± 11.97	7.047, **10^–4^**
Ablation 1											
PPV	82.72	75.38	75.00	100	89.87	95.45	82.43	94.37	87.18	86.93 ± 8.81	0.669, 0.552
NPV	92.98	73.24	72.37	100	95.65	88.46	84.29	88.89	93.94	87.76 ± 9.61	2.618, **0.031**
TPR	93.06	72.06	70.83	100	95.95	87.50	84.72	88.16	94.44	87.41 ± 10.19	2.577, **0.033**
TNR	80.56	76.47	76.39	100	89.19	95.83	81.94	94.74	86.11	86.80 ± 8.68	0.308, 0.766
Kappa	73.61	48.53	47.22	100	85.14	83.33	66.67	82.89	80.56	74.22 ± 17.42	1.897, 0.094
Ablation 2											
PPV	83.13	69.62	75.36	100	90.12	89.23	83.78	88.24	91.30	85.64 ± 9.01	1.942, 0.088
NPV	95.08	77.19	73.33	98.67	98.51	82.28	85.71	80.95	88.00	86.64 ± 9.20	2.213, 0.058
TPR	95.83	80.88	72.22	98.65	98.65	80.56	86.11	78.95	87.50	86.59 ± 9.43	1.881, 0.097
TNR	80.56	64.71	76.39	100	89.19	90.28	83.33	89.47	91.67	85.07 ± 10.27	1.427, 0.191
Kappa	76.39	45.59	48.61	98.65	87.84	70.83	69.44	68.42	79.17	71.66 ± 16.94	2.938, **0.019**
Ablation 3											
PPV	74.42	69.14	66.25	100	86.67	85.48	74.36	83.78	86.49	80.73 ± 10.58	5.884, **10^–4^**
NPV	86.21	78.18	70.31	96.10	87.67	76.83	78.79	82.05	88.57	82.75 ± 7.71	4.510, **0.002**
TPR	88.89	82.35	73.61	95.95	87.84	73.61	80.56	81.58	88.89	83.70 ± 7.42	2.953, **0.018**
TNR	69.44	63.24	62.50	100	86.49	87.50	72.22	84.21	86.11	79.08 ± 12.77	5.040, **0.001**
Kappa	58.33	45.59	36.11	95.95	74.32	61.11	52.78	65.79	75.00	62.78 ± 17.75	6.560, **10^–4^**

Bold significance values indicate *p*-values below 0.05.

### Comparison of computational time

Finally, to demonstrate that the new method is useful for BCI applications, we tested its computational efficiency. As BCIs require not only high decoding accuracy but also relatively fast parsing of the intended motion, a speedy classification is important. We used a 2.67 GHz i5-M480 processor with 4 Gb RAM to analyze the classification speed.

Runtime profiling of the algorithm took 366.91 ± 51.29 s for the entire assessment. While this is nearly 2.8 orders of magnitude longer than the similar contender algorithm (SBS-FBCSP) which took only 8.05 ± 3.02 s) this test compared both the feature selection/validation and classification. As the feature selection is only required for the model training, a comparison of the online classification alone showed that our algorithm is on par with competing algorithms that report their computational efficiency (Zhang et al., 2018). Namely, it is within 3 s from the SBS-FBCSP algorithm (n.s.). Together with the improved classification accuracy, we argue, the sacrifice in computational efficiency still renders our method ideal for BCI applications, and comparable to leading benchmark algorithms.

## Discussion

We evaluated the performance of a novel neural decoding algorithm, which used both temporal and spectral EEG signals, in predicting a motor action planned by subjects. Our algorithm showed increased accuracy of 2.06–4.94% above benchmark algorithms using two different standard dataset (Tables 1, 2).

The main differences between our method and the state-of-the-art algorithms tested were the inclusion of both temporal and spectral signals as inputs, and the extended features selection process. We suggest that these changes are key drivers of the performance improvement. Namely, we propose that the combined feature sets capture information that amplifies the variance within trials of a single individual and therefore increase the performance. To explore this hypothesis, we performed an ablation study where we hindered the inputs by altering the set of features included in the analyses and showed that the decoding accuracy decreased by an average of 3.88%. Even with the drop in accuracy, our algorithm was on par with state-of-the-art algorithms. As a sanity check, our results show that a decrease of the number of temporal features to a single feature yielded performance that was parallel to that of contender methods which only used spectral features.

Given that our method relies on the choice of a free parameter, *m*, we also tested the algorithm’s robustness to the parameter selection and showed that the results remain consistent (Tables 7, 8). Further, given that the choice of temporal window size was done arbitrarily in previous works, we tested a range of windows as well as a numbers of frequency bands permutations and showed that the results remain within ±1.49% for dataset 1 and ±1.74% for dataset 2, indicating that the decision is valid and reasonable.

In dataset 2, two algorithms outperformed our implementation. Both algorithms used an approach that deviated from traditional feature extraction methods. One algorithm used multi-scale CNN as a mechanism for the feature selection and the other used montage irregularities. These algorithms’ average performance increase was 8.03% (0.85 standard deviations) above our method. Given that both our algorithm and the contender ones show an effective deviation from traditional feature extraction methods, we suggest that a focus on improving this part of the MI classification process may be key to the success of novel methods.

### Contribution

In addition to proposing a new algorithm that implements various suggestions from a large corpus of prior works and yielding an improved performance, we also demonstrate the robustness of the method in multiple ways. We estimate the algorithm on two different datasets (allowing for generalizability of the implementation) and identify dominant parameters driving the performance. We situate the work in the context of existing algorithms and suggest that the process of feature extraction followed by independent classification maximizes the performance yield. Using inputs that are not traditionally considered for MI the expansion of classification set affords the algorithm a richer idiosyncratic noise minimization and tuning option. We show that the algorithm is offering an improvement without considerable hyperparameters tuning. Finally, we show that expanding the input set and the processing steps does not come at a significant cost with respect to decoding speed. The proposed algorithm can show generalized improvement in near real-time on consumer-grade computation tools, making it a viable method for future implementations by practitioners (Massaro et al., 2020) as well as academics (Cerf et al., 2007).

### Prior works

Our method is not the first to consider the multi-modal structure of EEG signals along with a dedicated classification tool during MI. For example, previous work (Deng et al., 2021) has combined temporally constrained group LASSO with CNN to interpret the underlying mechanisms driving the successful EEGNet decoding (Lawhern et al., 2018). Similarly, a framework for time frequency CSP smoothing was recently implemented to improve EEG decoding performance through ensemble learning (Miao et al., 2021). Both those methods focused on selecting CSP features by ranked weight. Conversely, our method incorporated the temporal features selection using a neural network. The neural networks classifiers were previously suggested as an extension of the establish body of works for MI tasks (Bhatti et al., 2019), yet were not implemented. Our work suggest that the non-linear feature selection provided by the network yields notable performance increase.

Focusing on the neural network implementation, it is noteworthy that a number of classifiers were proposed as variations on the method we used. Due to the recent developments in deep learning algorithms a majority of the methods proposed focused on CNN for the motor classification (Lawhern et al., 2018; Xu et al., 2018; Amin et al., 2019; Dy et al., 2019; Zhang D. et al., 2019; Zhang R. et al., 2019; Zhao et al., 2019; Chen et al., 2020; Ha and Jeong, 2020; Jia et al., 2020; Lian et al., 2021; Musallam et al., 2021). Specifically, Sakhavi et al. (2018) utilized CNN with temporal data, spectral data, and combination of these data to show a notable improvement in the classification performance compared to benchmark methods. Similarly, Dai et al. (2019) and Sun et al. (2020) showed that adoption of Squeeze-and-Excitation networks (Hu et al., 2018) in the CNN architecture improved the classification further because they accounted for the inter-dependencies among the EEG channels in the calibration of the spectral responses. In parallel, Zhang D. et al. (2019, 2020) and Chen et al. (2021) have implemented attentional mechanisms within the neural network architecture to benefit from the temporal dynamics of subject-specific signal properties. In line with these methods, Zhang D. et al. (2019) and Jia et al. (2020) deployed a multi-branch strategy that benefited from the idiosyncratic temporal-properties of different subjects by utilizing complementary networks. Applying the same logic to spatial-temporal signals, Li Y. et al. (2019) used CNN to capture mixed-scale temporal information and improve the decoding accuracy. In addition to improving the input signal features selection, novel methods have focused on bettering the feature discrimination and selection strategies (Yang et al., 2021) and the data augmentation tools (Li Y. et al., 2019; Yang et al., 2021). Specifically, investigating the input features further, Jin et al. (2021) have introduced time filter to a task-related component analyses method that enhanced the signal detection. The works used singular value decomposition to suppresses the general noise and increase the classification accuracy. The method was implemented on steady-state visual evoked potential based BCIs which are different than our data, but it is likely that the method will be useful for our data as well because of the similarity in decoding performance. Beyond similarity in noise reduction, previous works have also improved the feature selection optimization as we did. Jin et al. (2020) implemented feature selection based on the Dempster-Shafer theory which considers the distribution of the features and found the optimal combination of CSPs that minimized the influence of non-stationarity in the signal. Similar to our implementation, this method took into account the inherent defects of CSPs. Further, the work proposed an investigation of the temporal-spectral feature binning for the BCIs similar to the way bins were integrated into the sequential backward feature selection process in our work. Additionally, Jin et al. (2019) have proposed a correlation-based channel selection combined with regularized CSP (RCSP) as a way to improve the classification accuracy. The method seems to align with ours in its performance despite the fact that the RCSP does not consider both the temporal and spectral properties of the MI. The inclusion of both temporal and spectral feature types is suggested in the work as a future endeavor to be investigated. Completing the previous work, Li et al. (2018) have reported that using multiple modality inputs (in their work, both audio and visual signals) to enhance the representation of incoming signals yield increased accuracy in action decoding task (in their case: decoding “crying” vs. “laughing”). The work suggests that in addition to richer signal, the multi-modal inputs afford comprehensive data that benefits from the internal correlation among features. Besides being analogous to our work in their approach to the decoding, these works suggest that rich (or even superfluous or noisy) data inputs can prove useful in classification improvement. While the data in some of the listed works are different (i.e., fMRI data, or different tasks data) we intuit that the methods could be used to improve our work toward an even greater accuracy in the MI decoding. Finally, the network architecture itself was optimized in several works. For example, LSTM and RNN were incorporated in the CNN with the intent to capture additional properties of the EEG signal segments (Ko et al., 2018; Zhang D. et al., 2019; Zhang R. et al., 2019; Lian et al., 2021). Together, all these methods have demonstrated the benefit of incorporating subject-specific temporal properties in the neural network and the advantages these data have in improving the decoding performance.

Since our method implements feature selection and subject-idiosyncratic inputs in the training, as well as further granular features breakdown along the one discussed here, we suggest that our proposed method benefits from the collection of previous advantages. Namely, as our method separated the feature selection process from the following classification task, we suggest that the two-stage process, which enabled the reduction of features number, is one of the significant drivers of the performance increase.

### Comparison to leading contender algorithms

Comparing our method to an algorithm that uses similar routines (SBS-FBCSP) showed average increased performance of 20.22% (19.28% for dataset 1, and 21.15% for dataset 2). Similarly, a comparison to two other algorithms that share key characteristics with ours, albeit with less direct alignment in the protocol (SFBCSP and SFBCSP-MT) showed an incremental performance increase for our proposed method.

While the SBS-FBCSP was the algorithm conceptually closest to ours and, therefore, the subject of the main comparison, it is useful to highlight some of the similarities and differences between our method and other popular classification protocols.

Examining the notable similarities and differences between our method and 15 methods tested with dataset 1 (Table 10) or 21 tested with dataset 2 (Table 11) we note the main difference being the type of features selected as inputs, and the separation of the feature selection and generation steps.

**TABLE 10:** Comparison of contender algorithm implemented with dataset 1.

	Method	Similarity	Difference
1	DNN	Combined CSP with neural network	Did not use both temporal and spectral information
2	KPCA-CILK	Applied a conformal transformation to decrease the non-Euclidian characteristics of the signal while preserving the geometry	Did not use both temporal and spectral information
3	WOLA-CSP	Performed dynamic filtering of the EEG signal	Implemented the BCI on embedded platform, and did not use both temporal and spectral information
4	MEMDBF-CSP-LDA	Adopted common spatial pattern on reconstructed data from the multivariate empirical mode decomposition	Did not use both temporal and spectral information
5	JSTFD-LDA	Considered both temporal and spatial features by extending the CSPs	Did not use spectral information
6	nCSP-TSLR	Normalized and regularized the CSP to improve performance	Did not use temporal and spectral information
7	W-CNN	Took the wavelet time-frequency image of the EEG as input for the CNN	Both feature generation and selection done by the neural network
8	SS-MEMDBF	Utilized the MEMD to extract cross channel information as well as localize the specific frequency information	Did not use temporal information
9	CSP-FB-LOG	Adopted ensemble learning for feature selection from newly proposed feature extraction based on CSPs	Did not use temporal information
10	SW-LSR	Introduced the sliding windows techniques into the CSP	Did not use spectral information
11	EEGnet	Utilized depth-wise and separable convolutions in the CNN	Both feature generation and selection done by the neural network
12	R-MDRM	Regularized the covariance matrices using data from prior analyses of the EEG channels in small sample settings to reduce calibration time	Did not use both temporal and spectral information
13	SR-MDRM	Regularized the covariance matrices using data from other subjects in small sample settings to reduce calibration time	Did not use both temporal and spectral information
14	TSGSP	Adopted Group Lasso selecting the temporal-spectral common spatial pattern features in a multi-task learning manner. The selection of filter banks as well as temporal windows was similar to ours	Used SVM. Tuned three parameters rather than one
15	DCR-MEMD	Utilized the Gini and Maximum Information Coefficient for optimal channel selection as well as Multivariate Empirical Mode Decomposition for feature extraction	Did not use both temporal and spectral information

**TABLE 11:** Comparison of contender algorithm implemented with dataset 2.

	Method	Similarity	Difference
1	RSMM	Adopted a novel unified framework of robust matrix classifier as well as decomposition of EEG solved by alternating direction method of multipliers	Eliminated the effect of outlier and noise. Did not use temporal and spectral information
2	DLVA	Simultaneously incorporated temporal, spatial and spectral information using a CNN-variational autoencoder	Both feature generation and selection done by the neural network, and had no independent feature selection process
3	SGRM	Reduced the required training samples from target subject using auxiliary data from other subjects	Did not use temporal and spectral information
4	UDFS	Utilized an unsupervised feature selection strategy to select the potential CSP feature from multiple frequency bands	Did not use temporal information
5	SSD-SE-CNN	Proposed squeeze-and-excitation blocks embedded CNN to explore time-frequency features and classification	No independent feature selection process
6	WaSF ConvNet	End-to-end CNN using time-frequency and spatial information and using wavelet-like kernels to reduce the number of parameters	No independent feature selection process
7	CSP-Wavelet-LOG	Ensemble learning for feature selection using CSP-based feature extraction	Did not use temporal and spectral information
8	MAAN	CNN with multi-attention layers to capture the spatial property of the signal as well a domain discriminator to reduce the difference between sessions	Did not use spectral information
9	MTPP-EEGNet	Multi-layer temporal pyramid pooling approach incorporated into the CNN	Did not use spectral information
10	UDFS	Utilized an unsupervised feature selection strategy to select the potential CSP features from specific multiple frequency bands	Did not use temporal information
11	DJDA	Novel dynamic joint domain adaptation network based on adversarial learning strategy to learn domain-invariant feature representation using information from the source session	Did not use temporal and spectral information
12	SHNN	Consider both temporal and spectral information by segmenting the raw EEG into different windows and band-pass filtering the signal	Both feature generation and selection done by the neural network
13	TSLDA	Linear discriminant analysis included in covariance matrix	Covariance matrix did not use temporal and spectral information
14	DRDA	Deep representation-based domain adaptation to improve the classification performance on a single subject using information from multiple subject sources	CNN did not use temporal and spectral information for both the source and target domains
15	RF-DFFS	Dynamic feature selection strategy where EEG frequency domain features are selected one by one in a boosting protocol	Did not use temporal information
16	FDBN	Deep Belief Network classifier using the FFT features	Did not use temporal information
17	TSGSP	Group LASSO selecting the temporal-spectral CSPs in a multi-task learning manner. Selection of filter banks as well as temporal windows	Used SVM. Tuned three parameters rather than one
18	MMCNN	Multi-scale, multi-branch CNN to overcome the variation between time and subjects using convolution kernel in different sizes	Did not use both temporal and spectral information
19	WPD-STDF	New spatio-temporal discrepancy feature combined with frequency information	No feature selection strategy
20	CD-CNN	EEG data augmented using a circular translation strategy, followed by a central vector shift strategy to strengthen the discriminative power of the CNN	Did not use both temporal and spectral information
21	FBRTS	Fusing the features extracted from CSP as well as multiple time windows	Fusion strategy did not have feature selection strategy

### Limitations

The proposed decoding method suffers from a number of limitations that are driven by the extension of the temporal components. First, the method requires a priori intuition about the data in order to accurately choose the temporal segments. To prove the method’s superiority in datasets where no prior knowledge is available it would be useful to test either arbitrary datasets, or randomly selected temporal windows. If the method proves superior even with those selections, it will be regarded more robust.

Second, the algorithm has additional degrees of freedom that could be optimized with regards to the selection of hyperparameters. We elected to use the default ones operationalized by the Matlab implementation (Matlab version 2018a) without any additional tuning, but recognize that future work could focus solely on tuning those hyperparameters. Given the lack of theoretical justification for any alternative choice and given that the contender algorithms also used the default hyperparameters without additional emphasis on tuning, we did not deviate from this norm. Ideally, future work will yield theoretical reasoning for some of the tuning alternatives and thereby improve the algorithm’s performance as well as its usefulness for varying test cases outside of the MI implemented here.

Third, our method could be orders of magnitude slower in its initial computation training time than other methods. This means that usage of the method for BCIs that continuously update the feature set would either be challenging or require extensive computational resources. To overcome this challenge, one should investigate whether smaller time-window sizes (presumably yielding faster processing) could produce higher performance. Shorter time-window that maintain the high performance would elevate the usefulness of the algorithm.

Fourth, it is not clear whether the method would easily generalize to BCI tasks outside of MI. Specifically, because MI tasks are less likely to show the types of noise that pollutes active motor actions, the fact that our method shows superiority in one domain does not guarantee its success in others. We focused on implementing the method on MI tasks as these are the ones mostly implemented thus far and because of their ecological validity in the context of therapy and rehabilitation (Sokol et al., 2019). Implementing the method in other domains (i.e., language decoding) would validate the usefulness of the method further, or highlight the differences in the BCI uses.

### Future directions

Two research venues that directly extend our work are: i) the enhancement of the features selection granularity (while attempting to maintain the feature-classification performance), and ii) the generalization of the temporal features classification process. Specifically, as EEG and other biological signals are heavily dependent on combined temporal and spectral dynamics, usage of feature selection process with tools such as the recently proposed attention guided neural networks (Sun et al., 2019) may improve the ability to extract the appropriate features without a priori knowledge on the data. This would make the algorithm generalizable to other BCI inputs.

Further, as the majority of the benchmark algorithms we compared use neural networks for the full classification process (thereby effectively using all the available features without pre-selection) we suggest that amending the benchmark algorithms to focusing on the deep learning ones incorporating the two-step selection-classification process may increase the performance of all the benchmark methods.

It has not escaped our notice that as SVMs were previously shown to be superior with respect to feature classification (whereas deep learning networks were shown to be superior in BCI feature selection; Li Y. et al., 2019; Deng et al., 2021; Tiwari and Chaturvedi, 2021) a combination of both methods might improve our algorithm further and allow it to generalize to tasks outside of MI or motor control (i.e., non-verbal communication, language decoding, or parsing of thoughts).

## Conclusion

In this work we have shown that an algorithm which incorporates both temporal and spectral EEG inputs can yield high performance in recognizing which action was imagined by a subject. The algorithm uses SBS technique to reduce the number of inputs and to identify which inputs are less likely to be idiosyncratic across subjects. Once the input features are selected, a RBFNN is used for the classification of the action. We suggest that the method yields performance improvements compared to existing protocols primarily because the inclusion of the large subset of features reduces the individual noise idiosyncrasies within subjects. The suggested algorithm incorporates many of the benefits of the current corpus of state-of-the art BCI protocols and implements the improvements suggestion offered by numerous prior works. In line with these prior suggestion, the method could be applicable for other neural classification problems, modalities, and domains outside of the ones tested herein.

## Data availability statement

Publicly available datasets were analyzed in this study. These datasets can be found here: Leeb et al. (2008).

## Author contributions

GW conducted the analyses. Both authors wrote the manuscript and generated the final output.
